# Assault-related traumatic brain injury hospitalizations in Canada from 2010 to 2021: rates, trends and comorbidity

**DOI:** 10.1186/s40621-024-00486-5

**Published:** 2024-02-07

**Authors:** Shikha Saxena, Sarah Zutrauen, Steven R. McFaull

**Affiliations:** Public Health Agency of Canada, Ottawa, ON Canada

**Keywords:** Assault, Head injury, Concussion, Causes

## Abstract

**Background:**

Traumatic brain injury (TBI) is a major cause of morbidity and mortality globally. TBI is often associated with other physical or psychological issues resulting in high hospitalization costs. TBI incidence and recovery can vary with the external cause being intentional or unintentional. It is important to monitor the rates of TBI hospitalizations related to different external causes. This study examined the annual rate, comorbidity and length of stay associated with assault-related TBI hospitalizations and compare it with other external causes, by age and sex in Canada from 2010 to 2021.

**Methods:**

Discharge Abstract Database was used to extract cases of TBI (2010–2021). ICD-10-CA codes were used to classify all cases with TBI as per assault and other external causes (falls; transport; sport, physical activity and recreation; struck by). Additional variables, including age, sex, comorbidity and length of stay, were examined. Time trends were quantified using Joinpoint regression.

**Results:**

The average annual percent increase for all TBI hospitalizations from 2010 to 2021 was not significant at 0.1%. Females accounted for 35.8% of these TBI hospitalizations. From 2010 to 2021, assault-related TBI hospitalizations showed a significant annual decline of 4.1% for males and a significant increase of 1.2% for females. Increase in TBI hospitalizations related to falls showed an average annual percent increase of 1.4% for males and 2.2% for females. A significant decrease was observed for TBI hospitalizations related to the other three (transport, SPAR and struck by) external causes for both sexes from 2010 to 2021. Infants and children under 10 years of age had higher percentages of cases with comorbidities and higher length of stay for assault-related TBI hospitalizations.

**Conclusions:**

Assault-related TBI hospitalization rates decreased overall and among males, rates among females increased from 2010 to 2021. These results underscore the importance of targeted prevention efforts for TBI related to different external causes, age and sex, and continued surveillance to monitor the epidemiology of assault-related TBI.

## Introduction

Traumatic brain injury (TBI) has been recognized as the one of the leading causes of death and disability by world and national health experts (Fu et al. 2015; World Health Organization 2006; Rao et al. 2017). TBI is defined as “an alteration in brain function, or other evidence of brain pathology, caused by an external force” (Menon et al. 2010). The age-standardized incidence rates for TBI in 2016 were reported to be 369 per 100,000 population globally and 302 per 100,000 for Canada (James et al. 2019). Along with the high incidence rates, the burden of TBI on healthcare costs is significant because of the physical and the psychological comorbidity associated with TBI (Humphreys et al. 2013). Both preexisting and new onset comorbid conditions are reported to be common among individuals with TBI (World Health Organization 2006). TBI can result in permanent physical, psychological, cognitive and social dysfunction, influencing major personal and economic costs (Andelic et al. 2013). Owing to the complexity of TBI symptoms, TBI is often associated with high hospital length of stay and hospital resource utilization (Caplan et al. 2018; Masel 2015). In Canada, significant trends have been reported toward increasing age, comorbidity, injury severity and hospital length of stay over time from 2006 to 2010 for TBI hospitalizations (Fu et al. 2015). It is critical that the TBI hospitalizations are monitored by age, sex and comorbidity to provide valuable information regarding their burden on health care.

Within Canada, the overall hospitalization rates for TBI remained stable for young adults (15 to 24 years), but they increased for the elderly population (> 65 years) between 2006 and 2011 (Fu et al. 2015). TBI hospitalization rates from 2006 to 2017 in Canada showed an increase of 2% per year for females, while the rates for males were found to be stable (Public Health Agency of Canada 2020). In addition to age and sex, another key variable that explains the epidemiological trends of TBI and is vital to inform targeted prevention programs is the external cause of injury. TBI results from different external causes, which might be intentional (assault, suicide, self-harm) or unintentional (falls, transport, sports struck by/against and other). These external causes are associated with different levels of injury severity, health outcomes and the utilization of health resources during TBI recovery (Majdan et al. 2011; Asemota et al. 2013). As per the Centers for Disease Control and Prevention (CDC) 2021 report, falls and transport were the leading causes of TBI in 2017 in the USA (CDC 2016). In Canada, TBI hospitalizations resulting from falls showed a 29% increase for the elderly population (65 years and over), whereas the hospitalization rates for transport-related TBI showed a 28% decline for young adults (15–24 years) between 2006/2007 and 2010/2011 (Fu et al. 2015). A recent systematic review reported similar trends for Europe, where the proportion of TBI related to falls showed an increasing trend and those related to transport showed a decreasing trend over a period of the last 10 years (Brazinova et al. 2021).

Other important causes that are contributing to the burden of TBI across the globe are physical assault and violence. In addition to falls and transport, assaults were also reported to be among the leading causes of TBI cases in the USA between 2002 and 2010, with the most severe injuries requiring medical care and hospitalization (Frieden et al. 2015). Previous reports from Canada have shown that assault-related TBI are infrequent between individuals aged 2 and 14 years but began to rise at around 15 years of age, particularly for males (Public Health Agency of Canada 2020). Assault-related TBI hospitalizations were highest among 20–29 year olds at 22.6/100,000 population between 2006 and 2017 in Canada (Public Health Agency of Canada 2020). Assault-related TBI is reported to be a distinct clinical group with greater symptoms, disability and caregiver burden (Hanks et al. 2003; Schopp et al. 2006; Kim et al. 2013a). Although few studies examined trends in TBI hospitalizations related to assaults in Canada until 2017, no studies examined the comorbidity and length of stay associated with assault-related TBI at a national level. The current evidence needs to be supported by investigating more recent rates, trends and comorbidity for assault-related TBI hospitalizations in Canada in comparison with other main external causes of TBI (falls, transport, sports and struck by/against) (Langlois et al. 2006). Understanding the variation in TBI patterns over time is key to identify specific targets for prevention initiatives (Butcher et al. 2007). The objectives of this study were to: (a) examine the annual rate and trends of assault-related TBI hospitalizations and compare it with other external causes, by age and sex; (b) examine comorbidity and length of stay associated with assault-related TBI hospitalizations and compare it with other external causes, by age, in Canada from 2010 to 2021.

## Methods

### Data sources

This was a population-based descriptive study of TBI hospitalizations in Canada between 2010 and 2021. This study used the Discharge Abstract Database (DAD), obtained by the Public Health Agency of Canada through a data sharing agreement with the Canadian Institute for Health Information. This study covers the period between fiscal years 2010/2011 and 2021/2022. The DAD captures administrative, clinical and demographic information on hospital discharges (separations) and is reported by fiscal year (April 1 to March 31). Data are received directly from acute care facilities or from their respective health/regional authority or ministry/department of health. Facilities in all provinces and territories except Quebec are required to report. A DAD record can include up to 25 diagnosis fields which contain information on external causes, body regions and natures of injury and other information related to diagnoses. International Classification of Diseases and Related Health Problems, 10th Revision, Canada (ICD-10-CA) codes are used to classify reported diagnoses in the DAD (DAD Abstracting Manual 2021).

### Case extraction

Cases with TBI were extracted from the DAD using the ICD-10 (World Health Organization 2004) and the Canadian enhancement (ICD-10-CA) (International Statistical Classification of Diseases and Related Health Problems 2015). For this study, the TBI definition recommended for neurotrauma surveillance using administrative data in Ontario, Canada, has been used (Chen and Colantonio 2011). ICD-10-CA codes were used to classify all cases with TBI as per assault and other external causes. Assault-related cases were extracted using the classification by Fujiwara et al. (2012) that includes codes that described causes for the head injuries (Fujiwara et al. 2012). Cases with codes for poisonings or sexual abuse were excluded. The breakdowns for other external causes reported in this study focused on—falls; transport; sport, physical activity and recreation related (SPAR); and struck by/against (Langlois et al. 2006). The SPAR category classification in this study is non-standard and created at the Public Health Agency of Canada. It is composed mainly of codes from the other categories—falls, transport and struck by/against (Majdan et al. 2011). For example, falls related to SPAR, bicycling and off-road vehicles are grouped in the SPAR variable and not in the general falls or transport group. The codes are provided in Table 1. There were no changes made to the coding system during the study period. The identification of cases with TBI and their classification by external codes were done by searching all 25 diagnosis fields. Therefore, the TBI hospitalizations might have co-occurring diagnoses and external causes for the episode of care. TBI hospitalizations also include repeat visits.

**Table 1:** Codes to identify traumatic brain injury and external causes using ICD-10 diagnostic codes

Category	ICD-10 code
Traumatic brain injury	F07.2, S02.0, S02.1, S02.3, S02.7, S02.8, S02.9, S06.0–S06.9, S07.1, T90.2, T90.5
Assault	T74.1, T74.8, T74.9, Y07.0–Y07.3, Y07.8, Y07.9, Y00, Y01, Y04, Y08, Y09, Y29, Y30, Y33, Y34
Transport	V01–V09; V20–V29; V30–V39; V40–V49; V50–V59; V60–V69; V70–V79; V80.1–V80.7; V81–V85; V87–V89; V90.0; V90.1; V91.0; V91.1; V92.0, V92.1; V93.0, V93.1; V94.0, V94.1; V95; V97.0, V97.1, V97.3, V97.8; V99
Sport, Physical activity and Recreation	V10–V19, V80.0, V80.8, V80.9, V86, V90.2–V90.9, V91.5–V91.9, V92.2–V92.9, V93.2–V93.9, V94.2–V94.9, V96, V97.2, V98, W02, W09, W16, W21, W22, W51, W5107, W67–W70, W73, W74, X50, U99
Falls	W00, W01, W03–W08, W10–W15, W17–W19
Struck by/against	W20; W22.08, W22.09; W50; W51.08, W51.09; W52

Comorbidity was measured using ‘Comorbidity count code’ from the DAD. It provides the number of significant comorbidity diagnoses associated with a case, as follows: 0—no significant comorbidity diagnosis; 1—one significant comorbidity diagnosis; 2—two significant comorbidity diagnoses; 3—three or more significant comorbidity diagnoses. A comorbidity was defined as a condition that coexists with the most responsible diagnosis at the time of admission or that subsequently develops and meets one or more of the three criteria for significance (Institute and of Health Information 2022). The criteria for significance are that the condition 1—requires treatment beyond maintenance of the pre-existing condition, 2—increases the length of stay by at least 24 h and 3—significantly affects the treatment received (Institute and of Health Information 2022).

Length of stay was measured using the variable, acute length of stay, which is the total number of days the patient was in acute inpatient care per each visit. Time spent in alternate level care (ALC) was excluded as ALC refers to when a patient is occupying a bed in a facility but does not require the intensity of resources or services provided in that care setting.

### Data analyses

#### Hospitalization rates for TBI

Descriptive statistics were used to describe TBI hospitalizations related to assaults and other external causes between 2010 and 2021. Age-standardized rates per 100,000 were directly standardized to the 2011 Canadian population. Age group (0–11 months, 1–9, 10–14, 15–19, 20–29, 30–39, 40–49, 50–59, 60–69, 70 years and over), sex (male and female), fiscal year, external cause of injury, comorbidity and length of stay were used to describe the cases. Cases where sex was coded as “other” were excluded because of low counts. Gender-based analyses could not be conducted as there was no available variable at the time of this analyses. Age-specific rates per 100,000 population were calculated using Statistics Canada population estimates over the study period.

To compare comorbidity associated with different age groups between external causes, log-linear modeling was used. Log-linear modeling is used for categorical variables that require three- or higher-dimensional tables. This study used log-linear models to examine whether interaction of comorbidity and age group was different across external causes. Two-way ANOVA was used to compare length of stay among age groups between external causes. In case the assumption of normality was violated, log transformation was used.

Age-standardized rates were used for trend analyses. Trends were quantified using joinpoint regression software tests (Jointpoint regression program 2018) that locate inflection points (joinpoints) and calculate whether the annual percent change (APC) of each identified segment was significantly different from zero at the alpha = 0.05 level and produces a 95% confidence interval (CI). The weighted average (AAPC) was also calculated for the entire time span. Confidence intervals have been reported for the APCs and AAPCs.

SAS EG 7.1 (SAS Institute, Inc., Cary, NC, USA) was used for all descriptive analyses and to calculate age-specific rates. Joinpoint regression program version 4.7.0.0 (SEERStat, NCI, Bethesda, MD, US) was used to analyze TBI trends by sex and fiscal year. A *p* value of less than 5% was considered significant.

## Results

A total of 237,514 TBI-related hospitalizations were reported in Canada (excl. Quebec) between 2010 and 2021. The overall crude rate of TBI hospitalizations increased from 64.4 to 73.2 per 100,000 from 2010 to 2021. Females accounted for 35.8% of these TBI hospitalizations. Individuals 70 years of age and above accounted for 40.6% of the TBI hospitalizations. 89.8% of the total TBI hospitalizations had at least one of the external causes from the five categories (assault, falls, SPAR, transport, struck by/against). 7.6% of the TBI hospitalizations were related to an assault. The highest percentage of TBI hospitalizations (57.0%) were related to falls, followed by transport (15.0%) and SPAR (8.6%). Struck by/against was related to 2.6% of TBI hospitalizations.

### Trends in TBI hospitalizations

**Fig. 1:**
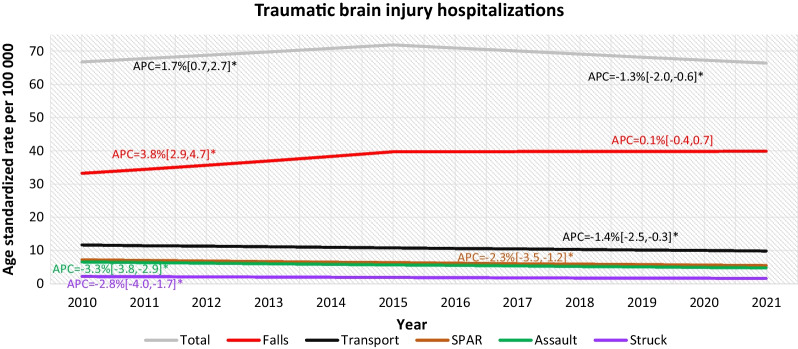
Age-standardized traumatic brain injury hospitalization rates per 100,000 population, by external cause, both sexes, all ages, Discharge Abstract Database, 2010–21 Canada (excluding Quebec). *APC* Annual Percent Change, *SPAR* Sports, Physical Activity and Recreation. *Represents significantly different from zero at the *α* = 0.05 level

Figure 1 shows the trend of overall age-standardized rates for all TBI hospitalizations and by external cause. The overall rate for all TBI hospitalizations showed a significant increase of 1.7% annually from 2010 to 2015 and a significant decrease of 1.3% annually from 2015 to 2021. The average annual percent increase for all TBI hospitalizations from 2010 to 2021 was 0.1%, which was not significant. The rates for TBI hospitalizations related to all external causes except falls showed a steady significant decline from 2010 to 2021. TBI hospitalizations related to falls showed a significant increase of 3.8% annually from 2010 to 2015 followed by no significant change until 2021.

Table 2 shows the age-standardized rates by sex and external cause. Males showed higher rates of TBI hospitalizations for all external causes. The rate for all TBI hospitalizations for males did not show any significant change between 2010 and 2016, but showed a significant annual decline of 1.8% from 2016 to 2021. For females, the rate for all TBI hospitalizations showed a significant 3.3% annual increase from 2010 to 2015 and a significant annual decrease of 1.0% from 2015 to 2021. The average annual percent change in the rates of all TBI hospitalizations for females was a significant increase of 0.9%. Males and females showed similar trends for TBI hospitalization related to all external causes except assault. Assault-related TBI hospitalizations showed a significant annual decline of 4.1% for males from 2010 to 2021, whereas females showed a significant annual increase of 1.2% from 2010 to 2021. Increase in TBI hospitalizations related to falls showed an average annual percent increase of 1.4% for males and 2.2% for females. A significant decrease was observed for TBI hospitalizations related to the other three (transport, SPAR and struck by/against) external causes for both sexes from 2010 to 2021.

**Table 2:** Age-standardized traumatic brain injury hospitalization rates per 100,000 population, by external cause and sex, Discharge Abstract Database, 2010–21 Canada (excluding Quebec)

Year	Total		Falls		Transport		SPAR		Assault		Struck by/against	
	Male	Female	Male	Female	Male	Female	Male	Female	Male	Female	Male	Female
2010	88.24	43.13	38.76	26.18	15.04	7.46	10.80	3.36	10.91	1.32	3.07	1.15
2011	88.68	44.57	39.90	27.54	14.84	7.49	10.51	3.31	10.46	1.33	2.98	1.12
2012	89.13	46.05	41.09	28.96	14.64	7.52	10.23	3.26	10.03	1.35	2.89	1.10
2013	89.59	47.58	42.30	30.46	14.44	7.55	9.95	3.22	9.61	1.37	2.80	1.07
2014	90.04	49.17	43.55	32.03	14.25	7.59	9.69	3.17	9.21	1.38	2.71	1.05
2015	90.50	50.80	44.84	32.22	14.05	7.62	9.43	3.13	8.83	1.40	2.63	1.02
2016	90.96	50.30	46.17	32.41	13.87	7.65	9.18	3.08	8.47	1.42	2.55	1.00
2017	89.29	49.79	45.97	32.59	13.68	7.69	8.93	3.04	8.12	1.43	2.47	0.98
2018	87.65	49.30	45.77	32.78	13.49	7.23	8.69	3.00	7.78	1.45	2.39	0.96
2019	86.04	48.81	45.57	32.97	13.31	6.81	8.46	2.95	7.46	1.47	2.32	0.93
2020	84.46	48.32	45.37	33.16	13.13	6.41	8.23	2.91	7.15	1.49	2.25	0.91
2021	82.91	47.84	45.17	33.36	12.95	6.04	8.01	2.87	6.85	1.51	2.18	0.89
APC	2010–16 0.5 (− 0.6, 1.7) 2016–21 − 1.8* (− 3.2, − 0.4)	2010–15 3.3* (2.6, 4.1) 2015–21 − 1.0* (− 1.5, − 0.5)	2010–16 3.0* (2.2, 3.8) 2016–21 − 0.4 (− 1.3, 0.5)	2010–14 5.2* (3.0, 7.3) 2014–21 0.6 (− 0.2, 1.3)	2010–21 − 1.3* (− 2.3, − 0.4)	2010–17 0.4 (− 1.8, 2.8) 2017–21 − 5.9* (− 11.0, − 0.4)	2010–21 − 2.7* (− 3.7, − 1.6)	2010–21 − 1.4 (− 3.1, 0.3)	2010–21 − 4.1* (− 4.7, − 3.6)	2010–21 1.2* (0.4, 2.9)	2010–21 − 3.1* (− 4.0, − 2.1)	2010–21 − 2.3 (− 4.6, 0.1)
AAPC	2010–21 − 0.6 (− 1.3, 0.2)	2010–21 0.9* (0.6, 1.3)	2010–21 1.4* (0.9, 1.9)	2010–21 2.2* (1.5, 3.0)	–	2010–21 − 1.9 (− 3.9, 0.2)	–	–	–	–	–	–

*SPAR* Sports, Physical Activity and Recreation, *APC* Annual Percent Change, *AAPC* Average Annual Percent Change

*Represents significantly different from zero at the *α* = 0.05 level

### Age-specific rates by external cause

**Fig. 2:**
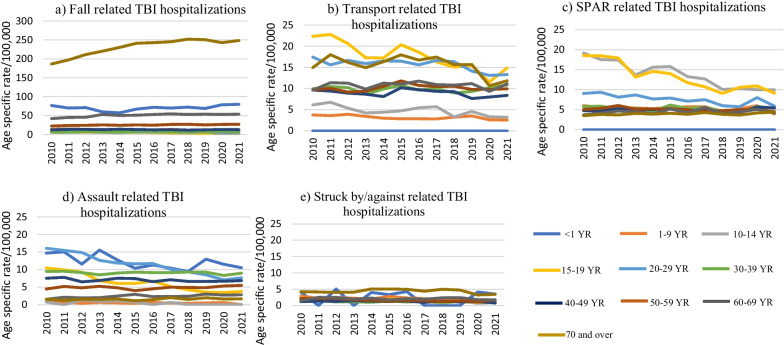
Age-specific traumatic brain injury hospitalization rates per 100,000 population by external cause, both sexes, Discharge Abstract Database, 2010–21 Canada (excluding Quebec). *TBI* Traumatic Brain Injury, *SPAR* Sports, Physical Activity, and Recreation

Figure 2 shows age-specific rates for TBI hospitalizations due to different external causes. Infants (< 1 year) and individuals aged 20–29 years had highest rate for TBI hospitalizations related to assaults. Fall-related TBI hospitalizations were highest among those aged 70 years and above, followed by infants. SPAR-related TBI hospitalizations were highest among 10–19-year-old age group. Transport-related TBI hospitalizations were highest among 15- to 29-year-olds and those aged 70 years and above. TBI hospitalizations related to struck by/against causes had the lowest rate among all external causes, with infants and those aged above 70 years of age showing the highest rates.

### Comorbidity and length of stay

**Fig. 3:**
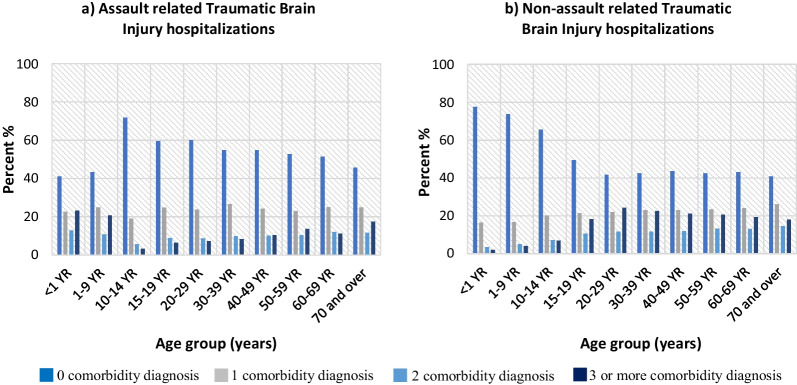
Percentage of traumatic brain injury hospitalizations with comorbidity diagnoses by age group, both sexes, Discharge Abstract Database, 2012–21 Canada (excluding Quebec)

Comorbidity was analyzed using counts of comorbidity diagnoses associated with each TBI hospitalization. This variable was added to DAD in 2012; therefore, the data for comorbidity analyses describe hospitalizations between 2012 and 2021. Among the TBI hospitalizations between 2012 and 2021, the information was missing for comorbidity for 9.0% (n = 18,297) of the cases. As the trends and percentages for the comorbidity variable by age was comparable among unintentional causes, the analyses were combined for SPAR, transport, falls and struck by/against as the non-assault category. TBI hospitalizations with at least one assault-related cause were included among the assault category. TBI hospitalizations where both assault- and non-assault-related causes were present were included among assault category. Figure 3 shows percentages of TBI hospitalizations with comorbidity diagnoses for assault and non-assault categories, by age group, for 2012–21. Log-linear modeling [Count = age group (10 categories) + comorbidity (4 categories) + external cause (2 categories, assault and non-assault) + age group*comorbidity*external cause] revealed significant interaction between age groups, comorbidity and external cause [*χ*^2^ (*df* = 27, *N* = 184,497) = 762.6.5, *p* < 0.0001].

Infants and children under 10 years of age showed higher percentages of 1 or more comorbidity diagnoses for assault-related in comparison with non-assault (SPAR, transport, falls, struck by/against)-related TBI hospitalizations. Individuals > 14 years of age showed higher percentages of 2 or more comorbidity diagnoses for non-assault-related when compared to assault-related TBI hospitalizations. Individuals 70 years and above showed comparable trends for the number of comorbidity diagnoses for assault and non-assault categories.

Log transformation of the mean length of stay was done to meet the normality requirement. Table 3 shows the log-transformed values of mean length of stay in days for TBI hospitalizations related to assault and non-assault causes by age groups (Table 3). Two-way ANOVA showed significant effect of the interaction of age groups and external cause (assault, non-assault) on the mean length of stay [*F*(*df* = 9, *n* = 232,112) = 5.43, *p* < 0.0001]. Children under 10 years of age showed higher length of stay for assault-related than for non-assault-related TBI hospitalizations. Individuals aged between 10 and 69 years showed higher lengths of stay for non-assault-related TBI hospitalizations in comparison with those related to assault. Individuals aged 70 and over showed higher length of stay for both assault and non-assault categories.

**Table 3:** Descriptive statistics for logarithmic mean length of stay for traumatic brain injury hospitalizations, both sexes, Discharge Abstract Database, 2010–21 Canada (excluding Quebec)

Age Group (years)	Assault-related TBI				Non-assault-related TBI			
	Mean (days)	Std Dev	Q1	Q3	Mean (days)	Std Dev	Q1	Q3
< 1	1.91	1.01	1.1	2.64	0.54	0.83	0	0.69
1–9	1.72	1.37	0.69	2.64	0.57	0.9	0	1.1
10–14	0.77	1	0	1.1	0.76	1.02	0	1.39
15–19	0.89	1.02	0	1.39	1.26	1.25	0	2.08
20–29	1.02	1.06	0	1.61	1.49	1.27	0	2.4
30–39	1.14	1.09	0	1.79	1.52	1.25	0.69	2.4
40–49	1.27	1.11	0	1.95	1.58	1.23	0.69	2.4
50–59	1.44	1.18	0.69	2.2	1.64	1.21	0.69	2.48
60–69	1.63	1.2	0.69	2.4	1.71	1.19	0.69	2.48
70 and over	1.89	1.29	0.69	2.77	1.81	1.11	1.1	2.56

*TBI* Traumatic Brain Injury

## Discussion

This study provides information

 about the emerging trends and epidemiologic patterns for assault related TBI hospitalizations in Canada (excl. Quebec) in comparison with other external causes. Overall, the age-standardized rates for TBI hospitalizations increased by 0.1% from 2010 to 2021. The age-standardized rates for TBI hospitalizations did not show any significant change for males and increased for females by 0.9%. Females showed a higher percentage of increase than males for TBI hospitalization rates related to assault and falls. Falls were the leading cause for TBI hospitalizations. Age-specific rates for TBI hospitalizations by external cause revealed important differences in the trends. Infants and adults between 20 to 29 years of age had higher rates for assault-related TBI hospitalizations for both males and females, while transport-related TBI hospitalizations were higher for the 15 to 29 age group. Adults aged 70 years and over showed the highest age-specific rates for fall-related TBI hospitalizations. Infants and children under 10 years of age had higher percentages of cases with comorbidities and higher length of stay for assault-related TBI hospitalizations. Individuals aged 15 years and over had higher percentages of cases with comorbidities and length of stay for non-assault-related TBI hospitalizations.

Epidemiology of TBI in Canada and peer countries with comparable health systems is widely researched. However, it is difficult to compare these data due to variations in TBI definitions and case extraction methods. As per the TBI report released in 2020 by Public Health Agency of Canada, hospitalization rates for TBI between 2006 and 2018 ranged from 38.6/100,000 to 47.8/100,000 for females and from 80.0/100,000 to 84.2/100,000 for males. In our study, females continued to show an increasing trend with the TBI hospitalization rates ranging from 43.1/100,000 to 47.8/100,000 between 2010 and 2021. On the other hand, males showed a declining trend in the TBI hospitalization rates from 2010 to 2021 with the rates ranging from 88.2/100,000 to 82.9/100,000. As per the CDC 2021 report, the rate for TBI hospitalizations was reported to be 63.3/100,000 population in 2017 in the US (CDC 2016). Recent estimates from the Victorian State Trauma Registry that extrapolate incidence of TBI to the national context are 46 cases per 100,000 population, with males having a higher rate than females (Victorian State Trauma Outcomes Registry and Monitoring Group 2022). While the overall trends and rates for TBI hospitalizations might be comparable across countries, it is important to note the interaction among different epidemiological variables—age, sex and injury mechanism—in order to recommend prevention initiatives targeting specific populations.

TBI research in Canada had mostly emphasized sports and falls related injuries (Gordon and Kuhle 2022; Cusimano et al. 2020), however, recent published work has started exploring the epidemiology and recovery mechanisms have used population-based hospital administrative datasets in one province in Canada (Kim et al. 2013a, b; Kim and Colantonio 2008). Two studies suggested that the rehabilitation needs of assault related TBI are significant, and that they should be provided appropriate care in order to integrate back into the community (Kim et al. 2013a, b). Our study highlights important trends on assault related TBI hospitalizations by sex between 2010 and 2021. Males contributed to majority of the assault related TBI hospitalizations, however, assault related TBI hospitalization rates showed a significant decline of 4.1% from 2010 to 2021 among males. Females, on the other hand, showed a significant increase of 1.2% for assault related TBI hospitalizations from 2010 to 2021. Assault related TBI hospitalizations were the only category among all external causes where females and males showed opposite trends for TBI hospitalization rates. These trends point toward findings that should be interpreted with caution. For example, the overall rates for assault related TBI hospitalizations for both sexes show a significant decline of 3.3% between 2010 and 2021, which is driven because of declining rate among males. Similar findings were reported in a population-based study in Australia, where hospitalizations related to assault showed a decrease of 2.7% for males and an increase of 1.1% for females, though both of which were not significant (Seah et al. 2019).

In our study, it was found that the number of comorbidities and the length of stay for assault related TBI was significantly higher for infants and children under 10 years of age in comparison to non-assault injury mechanisms. This suggests higher severity and complexity of assault related TBI hospitalizations among infants and children under 10 years of age and emphasizes greater need for clinical and social services. Longitudinal evidence suggests that infants and children who sustain TBI early in life have been shown to have persistent consequences on developmental (neuropsychological, cognitive, academic) outcomes (Ewing-Cobbs et al. 1997; Keenan et al. 2019). In addition to more awareness and education initiatives, a lot of attention is being given to improve the diagnosis of assault related or abusive head trauma in infants. Diagnosis of assault related TBI among infants and young children is often difficult because they might not present with specific symptoms and the history of trauma is provided by the caregivers. Misdiagnosis of assault related TBI can result in severe medical complication or even death (Jenny et al. 1999). Therefore, timely diagnosis of assault related TBI in infants and children can prevent long term hospitalizations and disability.

Our study showed that the TBI hospitalization rates related to transport and SPAR declined for both sexes from 2010 to 2021, whereas the rates related to falls have remained steady since 2016 for males and 2014 for females. Similar trends were reported by the CDC report, 2021 when comparing trends for 2016 and 2017 for both males and females (Majdan et al. 2011). TBI can happen in a variety of settings and a declining hospitalization rate suggests the effectiveness of multiple prevention initiatives within sports and transport sectors, and improved awareness for fall prevention within and outside Canada. However, older adults (70 years and above) continue to have the highest number and rate of TBI hospitalizations. In this study, older adults also showed higher number of comorbidities and length of stay associated with TBI hospitalizations both for assault- and non-assault-related TBI. With the growing population of older adults in developed countries, the burden of TBI among the elderly on clinical and social services would continue to rise as well (Caplan et al. 2018).

## Conclusion

This study provides information on the epidemiological trends and patterns of TBI hospitalizations related to assault and other external causes in Canada (excl. Quebec) between 2010 and 2021. Overall, TBI hospitalization rates increased between 2010 and 2015 and decreased between 2015 and 2021. However, it is important to understand how the trends vary by age group, sex or external cause. Although assault-related TBI hospitalization rates decreased overall and among males, rates among females increased over the study period. Our analysis of comorbidity and length of stay data indicated differences between assault- and non-assault-related TBI. However, there is a need for future studies to examine the types of comorbidities associated with assault-related TBI. Children under 10 years of age, including infants, showed higher percentages of cases with comorbidities and higher length of stay for assault-related hospitalizations. Further research analyzing the trends and complexities of assault-related TBI among children in Canada is needed. The results presented in this study may help support injury prevention initiatives among key populations. Continued efforts are critical to the prevention of TBI and should be contextualized within age groups, sex, external causes and other socioeconomic population variables.

## Data Availability

Data are available from the corresponding author on reasonable request.

## Abbreviations

CDC: Centers for Disease Control and Prevention
DAD: Discharge Abstract Database
ICD-10-CA: International Classification of Diseases and Related Health Problems, 10th Revision, Canada
TBI: Traumatic Brain Injury
